# Detection of Closing Cracks in Beams Based on Responses Induced by Harmonic Excitation

**DOI:** 10.3390/s24010247

**Published:** 2023-12-31

**Authors:** Samrawit A. Tewelde, Marek Krawczuk

**Affiliations:** Faculty of Mechanical Engineering and Ship Technology, Institute of Mechanics and Machine Design, Gdansk University of Technology, ul. Gabriela Narutowicza 11/12, 80-233 Gdańsk, Poland; marek.krawczuk@pg.edu.pl

**Keywords:** closed cracks, cantilever beam, nonlinear contact, harmonic vibration, phase diagram, nonlinear characteristics

## Abstract

The non-linear contact model was chosen to simulate a closed crack in a cantilever beam. This study examines the shape and characteristics of the phase diagram of a cantilever beam with closed cracks. It investigates how various crack properties influence the geometry of the phase diagram and proposes a method for identifying cracks based on their features. The area of each closed curve in the phase diagram was determined using the pixel method. Based on the results, the contact model proved effective in simulating closed cracks and was sensitive to nonlinear closing cracks. The vibration responses of beams with different damage severities and positions exhibited distinct geometric features. The crack parameter was identified by locating the intersection of contour lines on the maps. According to numerical calculations, the phase diagrams for super-harmonic resonance seem to be more susceptible to changes in closed cracks with varied damage locations and severities. The wavelet transform was also employed to identify closed cracks using RMS signals, and the results were compared with those obtained from the phase diagram.

## 1. Introduction

Cracks in structures can seriously compromise structural integrity, leading to catastrophic failure. In recent years, researchers have been dedicated to examining crack behavior [1,2,3,4,5,6]. Cracks are classified into two types: open and closed. Closed cracks are difficult to detect, but they can significantly weaken structural integrity over time [7]. Various techniques, including eddy current testing, magnetic particle testing, and ultra-sonic testing, can be employed for crack detection in beams. These methods are primarily applicable to static structures and may not be suitable for identifying complex or internal structural damage, where the damage may not be readily visible or accessible for inspection [8]. In contrast, vibration-based methods of damage detection can be applied to a wide range of structures and provide valuable insights into the condition of the structure without the need for direct physical access [9,10,11]. Vibration-based methods for crack detection fall into two categories: linear and nonlinear approaches. Linear methods, such as monitoring modal properties and resonant frequencies, have lower sensitivity and require significant damage for effective detection. Nonlinear approaches, suitable for early detection, rely on crack-induced nonlinear behavior. The closing crack model, for instance, captures dynamic response changes due to crack edges intermittently making contact during vibration cycles.

Nonlinear methods identify damage through features such as sub and super harmonics, phase diagrams, and the emergence of nonlinear normal modes [12,13,14,15,16,17,18]. Nonlinear vibration features, including harmonic response, structural bifurcation characteristics, and non-linear modes, show promise in describing dynamic characteristics in the early stages of crack formation. However, these methods, particularly modeling the behavior of a cracked beam with knowledge of the crack location and depth, require further development. Higher harmonics can serve as indicators of closed cracks [19]. K. Czelusniak et al. [20] work on nonlinear vibro-acoustic modulation, and they have been investigating higher-order spectral analysis for structural damage detection. J. Prawin et al. [21] proposed a method for the localization of breathing cracks using single sensor measurement in both single and multiple breathing cracks. Wimarshana et al. [22] use entropy measures to detect cracks. M. Cao et al. [23] propose a nonlinearity-sensitive approach for the detection of “breathing” cracks. A new energy modulation effect (EME) phenomenon was reported, based on a new concept of quadratic Teager–Kaiser energy (QTKE). This method considerably enhances the hidden higher harmonics, allowing for the easy detection of breathing cracks.

In structural analysis, the nonlinear index most often used is the harmonic response; additional features, including bifurcation and nonlinear strength [24], have also been established. These indices can be utilized to identify and examine various types of damage or defects, providing valuable insights into the nonlinear behavior of structures. Cracks are frequently analyzed qualitatively using the phase diagram, which features orbits that are sensitive to variations in the mechanical properties of structures [25]. The results show substantially nonlinear dynamic properties and include displacement and velocity information. They are also a helpful tool for comprehending how the closed cracks behave. Phase diagram analysis has gained attention for its ability to capture nonlinear dynamic characteristics associated with closed cracks. Numerous studies [26,27,28,29] have demonstrated the effectiveness of phase diagrams in capturing nonlinear dynamic characteristics associated with closed cracks. A careful analysis of the geometric features of the phase diagram provides valuable insights into the location and severity of closed cracks in different structures. Phase diagrams, commonly used in nonlinear analysis, contain abundant vibration information. Phase diagram distortion is a qualitative nonlinear damage indicator and is a major nonlinear dynamic feature of cracked systems. Utilizing phase diagrams has shown promise in identifying and characterizing structural damage. Srinivas and Saptarshi [30] demonstrated the effectiveness of phase diagram analysis in crack detection for beams, emphasizing the distinctive patterns that emerge in the presence of structural damage. The research highlights the sensitivity of phase diagrams to changes in structural dynamics induced by the presence of cracks, establishing the foundation for advanced crack detection methodologies.

Advancements in wavelet coefficients, highlighted by [31], have simultaneously offered a unique perspective on detecting localized changes in signal frequency associated with closed cracks. The integration of wavelet coefficients into closed crack detection methodologies has introduced a new dimension of analysis. Wavelet transforms, known for their capability to reveal localized changes in signal frequency, offer a unique perspective on crack-induced alterations in structural dynamics. The continuous wavelet transform (CWT) approach, which was studied by Andreaus et al. [32], introduces damage detection, enhances crack detection accuracy, and mitigates boundary effects. Research by [33] has notably demonstrated the utility of wavelet coefficients in detecting closed cracks and discriminating their characteristics with high sensitivity. As per [34], commonly used dynamic identification methods face challenges due to the problem of incomplete identification. Consequently, multiple measurement points are required to obtain more accurate results. The damage identification method based on natural frequency is not accurate for determining the location of structural damage. In symmetrical structures, the relationship between the frequency and damage locations is not unique, adding to the challenge of accurate identification. The harmonic response is currently the most used nonlinear index for detecting structural damage. In addition to this method, there are promising crack-identification techniques based on structural bifurcation characteristics, nonlinear strength, and nonlinear modes. However, due to the complexity of nonlinear problems, further development is necessary for these methods.

Wavelet transform, increasingly used for crack detection, exhibits excellent properties in extracting slope discontinuity from noisy signals. This work reviews strategies such as continuous wavelet transforms (CWT) and emphasizes crack detection at the edge of the beam. The measurement resolution in wavelet analysis is a critical factor for accurately detecting cracks. Building upon the existing body of literature on phase diagram analysis and wavelet coefficients, this research aims to implement a comprehensive methodology for closed crack detection. However, to the best of our knowledge, the application of phase diagram analysis and wavelet coefficients for closed crack detection in cantilever beams remains an underexplored area. Most existing studies have focused on breathing cracks. Therefore, this study aims to investigate and compare the effectiveness of phase diagram analysis and wavelet coefficients in closed crack detection. Additionally, it seeks to provide a significant understanding of how these methods individually contribute to closed crack detection. Through a detailed analysis of the geometric features of the orbital inclusion area of the phase diagram, we propose a method for determining crack depth and location.

## 2. Analytical Investigation

### 2.1. Model of Geometry

**Figure 1:**
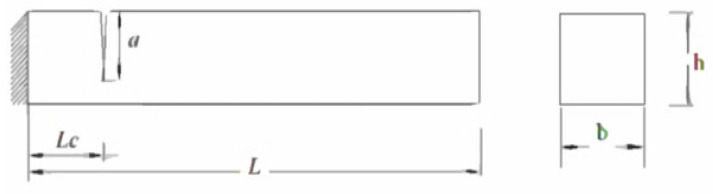
Model of a cantilever beam with crack.

The cantilever beam is made of steel with the left end fixed. Figure 1 shows that the length, height, and width of the beam are denoted by L, H, and B, respectively. A transverse breathing crack with a depth of ‘*a*’ is located at a distance ‘Lc’ from the fixed end of the beam.

For crack structure analysis, it is important to determine the resonant frequency since the structure is alert due to periodic load. The first step is modal analysis to determine the natural frequency of uncracked and cracked beams. For modal analysis, a cantilever beam of length 0.3 m, width 0.02 m, and depth 0.02 m is considered. In this case, the Mild steel beams were considered. Table 1 represents the properties of Mild steel. A breathing crack and transversal axis are present in the cantilever beam. The crack is positioned at three different locations denoted by P = *Lc/L*, where *Lc* is the distance between the crack location and the fixed end and L is the length of the beam. The values of P considered are 0.06, 0.5, and 0.7, with ‘*a*’ representing the depth of crack damage. The relative damage severity is expressed as s = *a*/h, where ‘h’ is the height of the beam, and values of 0.1, 0.3, and 0.5 are considered. The first mode of vibration is taken into consideration.

**Figure 2:**
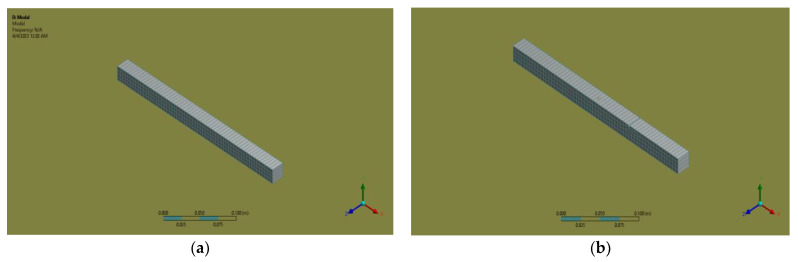
(**a**) Uncracked beam of Mild steel; (**b**) cracked beam of Mild steel.

The problem considered in this section is a cantilever beam with double cracks. Different crack depths and locations across the beam are considered, and their presence does not change the beam mass. Figure 2 shows the use of 20 nodes in the hexahedral element for both the uncracked and cracked beams.

### 2.2. Validation

During the initial stage of validation, the theoretical natural frequency of the uncracked cantilever beam was calculated. Calculations based on Pilkey [36] yield the natural frequency ($\omega_{n}$).

(1) $$\omega_{n}=\frac{\lambda^{2}}{L^{2}}\sqrt{\frac{EI}{\rho }}$$

By using Equation (1), $\omega_{n}$ of mode-1 is found as 185.6 cycles/s.

## 3. Finite Element Modeling and Analysis

The cracked beams’ natural frequencies are estimated using the finite element program, ANSYS [35]. A small region was first created and then extruded in the third direction to introduce small cracks with depths of 0.01 m at desired locations. These minor crack volumes were then subtracted from a large cantilever beam model, resulting in three-dimensional models with cracks along the top sides of the cantilever beam. The beam was modeled using a solid structure composed of 20 nodes and 186 hexahedral elements. This choice of element type allows for an accurate representation of the beam’s geometry and behavior. Finite element boundary constraints were applied to confine all degrees of freedom at the farthest left of the beam. The natural frequencies of the beams were determined using modal analysis. The initial validation involved analyzing a theoretical uncracked beam with a known natural frequency, which was then compared to the natural frequency obtained from the ANSYS analysis of the same uncracked beam.

### Closed Crack Model

**Figure 3:**
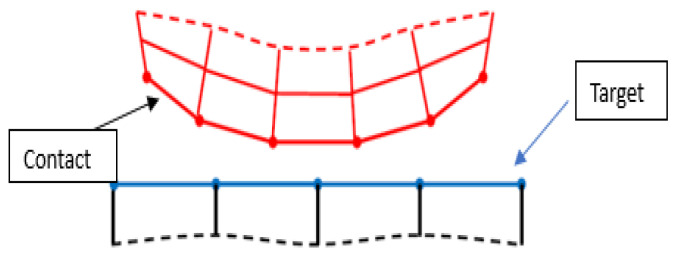
Nonlinear contact model.

The closed crack is a nonlinear feature, making it highly recommended to use a nonlinear contact modeling approach for its analysis [37,38], as shown in Figure 3. Two options for contact modeling are frictional and frictionless. Structural analysis generates contacts in pairs. Three available contact behaviors exist surface-to-surface, node-to-node, and node-to-surface. In a frictional contact model, the friction coefficient is greater than zero, leading to slipping along the contact surface when the tangential force reaches a certain threshold. In the frictionless contact model adopted in this paper, the friction coefficient is equal to zero, and the tangential force and loading path are independent. The calculation method is a pure penalty function and surface-to-surface contact is used, meaning that both the contact and the target are surfaces, with the contactor elements unable to penetrate the target surface but capable of doing so with the contactor surface.

## 4. Results and Discussion

### 4.1. Natural Frequency Cracked Beam

**Figure 4:**
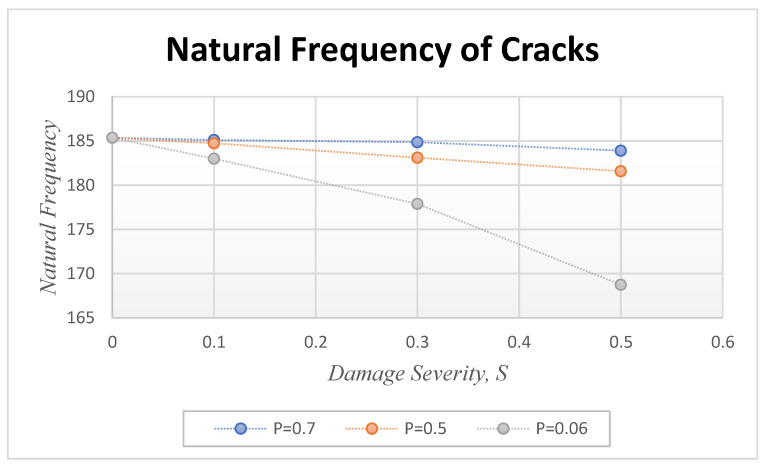
The natural frequency of different crack locations and severities.

Cracked beams at different crack locations and depths are considered in the analysis. Figure 4 depicts the natural frequency for different crack depths (S = 0.1, 0.3, and 0.5) and the crack locations (P = 0.06, 0.5, and 0.7). The table in the study indicates that the natural frequency decreases as the crack approaches the fixed end. Conversely, when the crack is farther from the fixed end, the natural frequency is closer to that of the intact beam. The depth of the damage also affects the natural frequency, as increasing the damage depth decreases the natural frequency at the same crack location. Figure 4 illustrates that the frequency increases are more significant as it approaches the free end. As the crack position moves away from the fixed end, the frequency drops and eventually reaches the frequency of the health beam.

### 4.2. Detection of Closed Cracks

When a cantilever beam with a closed crack is subjected to harmonic vibration, its response can be analyzed to detect the presence of the crack. The response is characterized by the amplitude and frequency of the vibration. By exciting the beam with a harmonic load at different frequencies and analyzing the response, the natural frequency and mode shapes of the beam can be identified. To verify the capability of the approach for detecting “closed” cracks subject to excitations at arbitrary frequencies, the beam is excited at resonance or non-resonance frequencies (ω1, 2ω1, and 1/2ω1 with ω1 denoting the first natural frequency of the beam). Harmonic load:

(2) F=Fosin⁡ωt

where *Fo* denotes the maximum value of the force, ω is frequency of the harmonic load, and *t* is time.

### 4.3. Effects of Crack Parameters on the Phase Diagram

**Figure 5:**
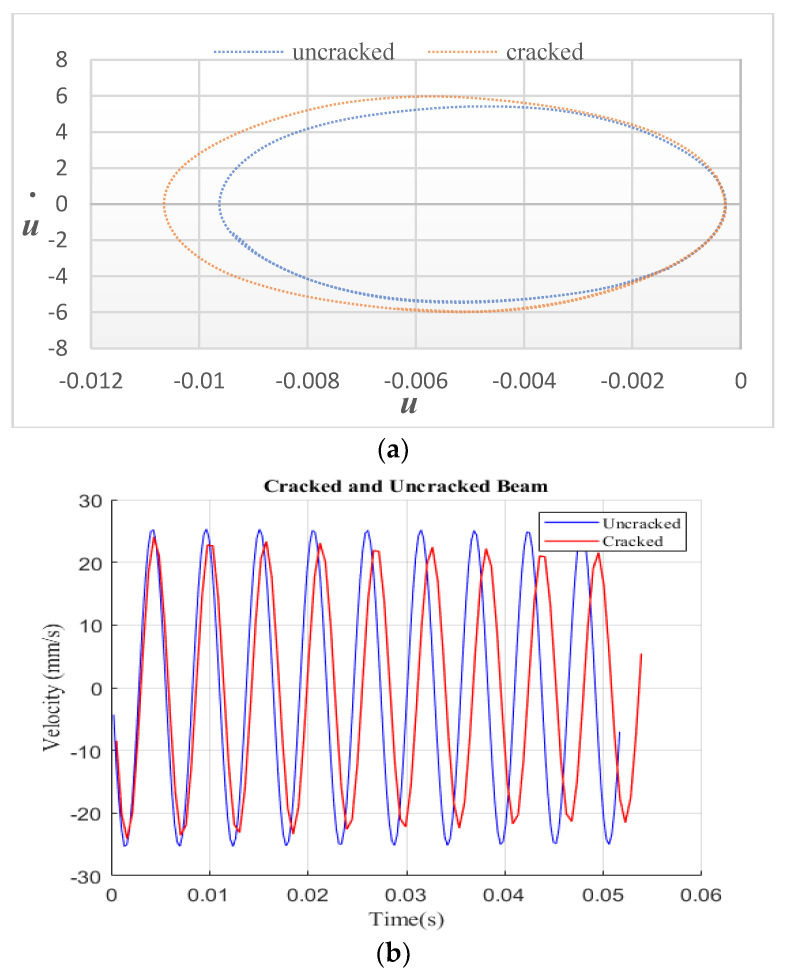
Harmonic vibration response: (**a**) intact and cracked beam; (**b**) velocity vs. time history.

This section discusses the effects of crack parameters on the phase diagram of a cantilever beam. The crack parameters considered include the crack depth and location. The phase diagram illustrates the relationship between displacement and velocity. Figure 5 displays the phase graphs for the cracked beam and the entire beam under the principal resonance frequency. Equation (2) was used to determine the excitation frequency at the beam edge’s end. According to the findings in Figure 5a, the uncracked beam is wholly circular in contrast to the cracked beam. Figure 5b demonstrates the acceleration with time response for a cantilever beam that is both uncracked and cracked. The results show that the crack has a significant effect on the phase diagram.

### 4.4. Effect of Crack Location

**Figure 6:**
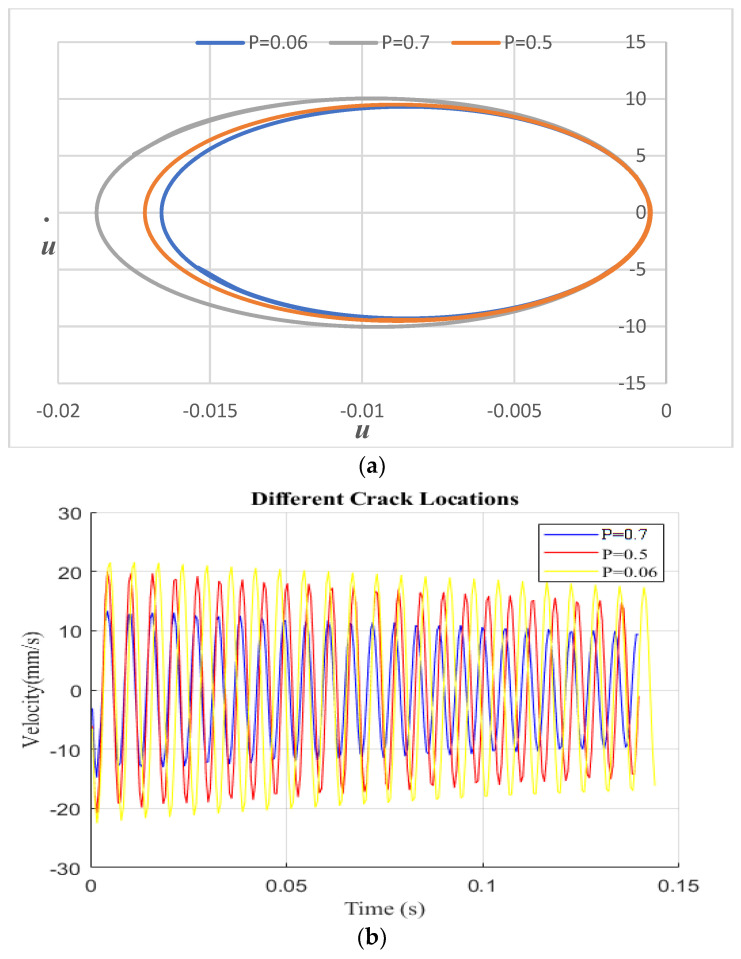
Harmonic vibration response: (**a**) different crack locations with constant crack depths and with excitation frequencies equal to primary resonance; (**b**) velocity vs. time history.

Figure 6 illustrates the impact of crack location on the cantilever beam with a fixed crack depth of S = 0.3. Three different crack locations, P = 0.06, 0.5, and 0.7, were considered. As shown in Figure 6, the crack becomes more visible and the displacement value increases when it is closer to the fixed end. Conversely, when the crack is located farther from the fixed end or closer to the free end, the displacement decreases and the damage becomes less apparent. Figure 6a displays phase diagrams for different crack parameters under the primary resonance frequency. The phase diagram exhibits a single loop as its path. These phase diagrams were created to visualize the impact of different crack locations on the behavior of the beam under the primary resonance frequency. The results show that crack location is an important factor to consider when analyzing the behavior of the cantilever beam’s phase response.

### 4.5. Effect of Crack Depth

**Figure 7:**
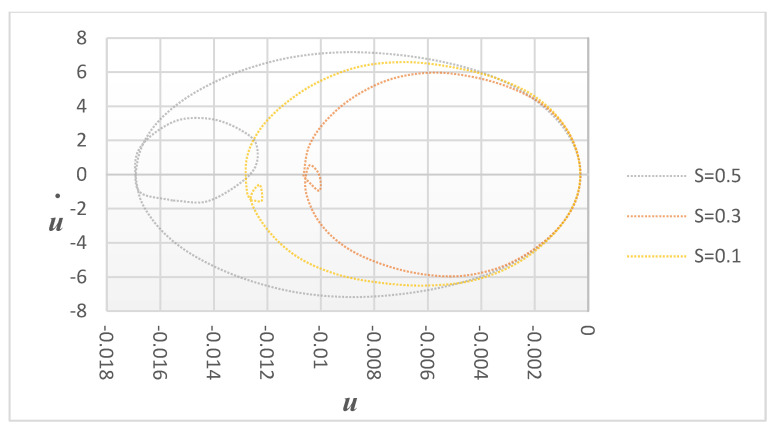
Harmonic vibration response: different crack depths with constant crack locations with excitation frequencies equal to the super-harmonic resonance frequency.

**Figure 8:**
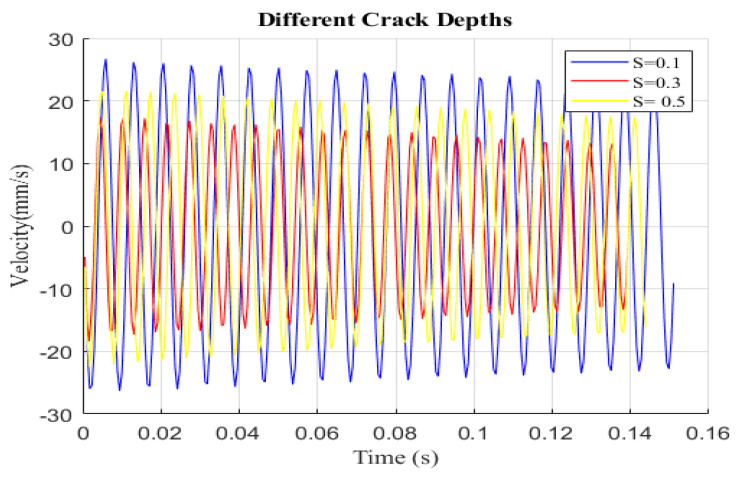
Velocity vs. time history of the cracked beam with different damage severities at *p* = 0.06.

Figure 7 demonstrates the effects of crack depth for specific crack locations by displaying variations in displacement versus velocity under various damage severities (s = 0.1, 0.3, and 0.5) at a fixed crack location of P = 0.06. As the damage severity increases, the small circle becomes more visible, indicating the significant effect of the crack depth on the structure. The phase diagram for different cracks is shown in Figure 7 and the excitation frequency is adjusted to the super-harmonic resonance frequency. Each curve has a large and tiny loop, as can be seen. This shows that both the damage location and severity are average, and it indicates that the phase diagram is an irregular single loop. This phase diagram shows a single loop for small crack depths but, for larger crack depths, two loops can be seen in the phase diagrams. This observation indicates that the vibration response of the cantilever becomes more pronounced as the crack depth increases. Figure 8 shows the velocity versus time for the same case.

## 5. Crack Parameter Detection Based on Phase Diagram Geometrical Features

The crack parameter detection process involves analyzing the geometric features of the phase diagram to determine the characteristics of the crack. By examining the shape, position, and area of the phase diagram, important information about the crack parameters can be inferred. Specifically, the phase diagram is divided into two regions: the phase area on the left side of a vertical dividing line and the full phase diagram area. These regions are labeled and analyzed to extract relevant information. The geometric features of the phase diagram, such as the shape of the dividing line and the area ratios, provide insights into the crack parameters. By comparing these features with known reference data or established models, estimates for the crack location and severity can be derived. This crack parameter detection technique based on the phase diagram analysis is valuable for real-time crack detection and characterization in engineering structures. Geometrical features of the phase diagram were utilized to detect the crack. Initially, the phase diagram displayed a vertical line that separated the two regions. The phase area on the left side of the dividing line was labeled as the crack area, while the entire phase diagram area was labeled as the healthy area. These regions were then extracted and their areas were calculated to identify the presence and extent of the crack.

The area of each closed curve on the phase diagram was determined using the pixel approach. To ensure the reliability of the crack parameter detection index, it is important to maintain a constant scale for the x-axis and y-axis intervals in the default drawing. The phase diagram image was captured within the default window of Matlab, ensuring an accurate representation. For the detection process, primary or super-harmonic resonance frequencies corresponding to various crack parameters can be used as the excitation frequency for the phase diagram. The crack depth and location ratios are kept unchanged during this analysis. This approach allows for a consistent comparison and evaluation of the phase diagram. To facilitate analysis, generating images that only include the phase diagram curves and dividing lines within the default graph window is recommended. This helps to focus on the essential features and minimize unnecessary visual elements.

### Vertical Segmentation Method

The vertical segmentation method is an approach used in crack parameter detection based on phase diagram analysis. This method can also broaden the variety of identifying indexes. By drawing a vertical line parallel to the y-axis, the phase diagram can be divided into two halves: the left and right sides. The ratio of the area to the right and left of the phase diagram is defined as the ratio parameter ‘n’.

**Figure 9:**
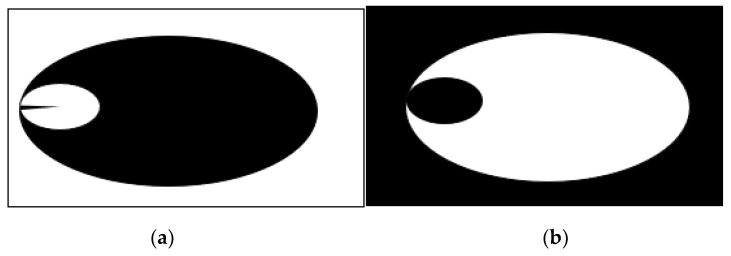
Double-loop phase diagram. (**a**) Smaller loop; (**b**) larger loop.

Under the excitation of the primary resonance frequency, the phase diagram curves of a cantilever beam remain mostly unchanged in the transverse direction, as shown in Figure 7. Consequently, the detection indexes for cantilever beams generated by super-harmonic resonance frequencies can be categorized into two types: single-loop phase diagrams and double-loop phase diagrams. Figure 9a illustrates the smaller loop section of a double-loop phase diagram, while Figure 9b shows the larger loop region. It is worth noting that, when subjected to the same excitation force, the amplitude of the beam generated by the super-harmonic resonance frequency is significantly lower than that of the primary resonance excitation frequency. Additionally, as the crack location and depth increase, the detection indexes gradually increase, indicating a higher level of damage. This method provides an additional dimension for crack parameter detection, enhancing the accuracy and reliability of the analysis.

Furthermore, the viability of the crack parameter detection procedure can be tested by analyzing the area ratios of the upper and lower curves in the phase diagram. The area ratio of the phase diagram’s right side to the left side is denoted as A(LR), while the area ratio of the downside to the upside is denoted as A(UD). These ratios provide further insights into the geometric features of the phase diagrams and can be used as detection indices for crack parameters.

**Figure 10:**
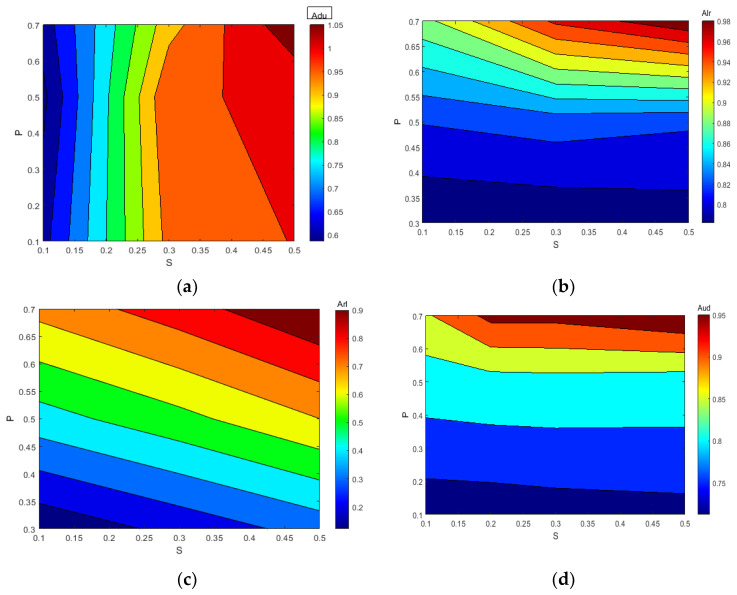
Variation in A(UD) and A(LR) with damage severity and location (**a**) S = 0.1 P = 0.5; (**b**) S = 0.3 P = 0.1; (**c**) S = 0.3 P = 0.7. (**d**) S = 0.3 P = 0.5.

Table 2 and Table 3 present the values of A(LR) and A(UD) for different damage parameters, allowing for a quantitative evaluation of the crack severity and location increase; the indices gradually rise, indicating a higher degree of damage. Figure 10 illustrates how index A(LR) increases while index A(UD) decreases with a crack depth or location increase. This provides a qualitative estimation of the degree of damage and the location of the cracks. The intersection point of the contour line formed by the two indexes is utilized to detect the crack accurately.

**Figure 11:**
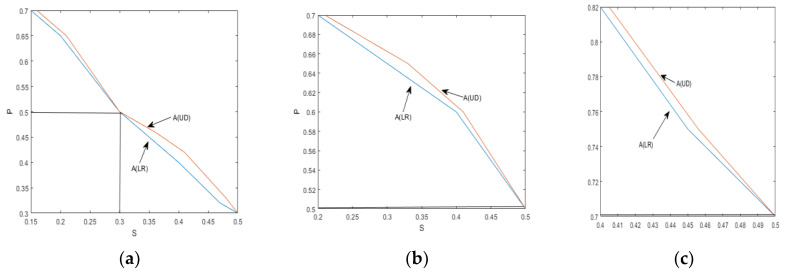
Crack parameter detection: (**a**) S = 0.3 P = 0.5; (**b**) S = 0.5 P = 0.5; (**c**) S = 0.5 P = 0.7.

**Figure 12:**
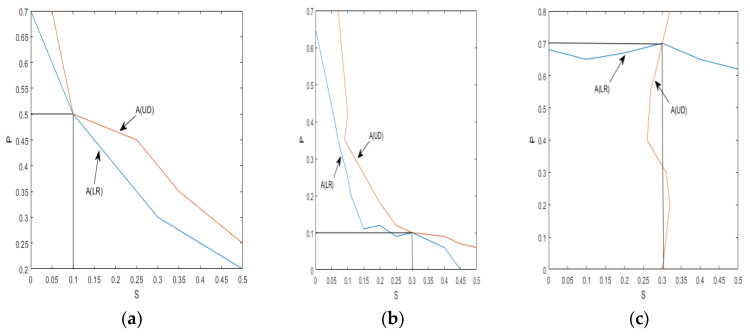
Crack parameter detection: (**a**) S = 0.1 P = 0.5; (**b**) S = 0.3 P = 0.1; (**c**) S = 0.3 P = 0.7.

Figure 11 and Figure 12 provide visual representations of how the indices A(LR) and A(UD) depend on the crack depth (S) and location (P). These figures illustrate the changing values of the indices as the crack parameter varies. Table 2 and Table 3 further support the observation that, as the damage depth or location increases, the index A(LR) steadily increases while the index A(UD) gradually decreases. This trend indicates that the level of damage and the position of the cracks can be qualitatively estimated based on the value of one of these indices. However, it is important to note that a single index alone cannot identify a specific crack, since multiple combinations of damage parameters can yield the same contour line with the same index values. Therefore, to precisely locate the crack, it is necessary to consider the intersection of the contour lines formed by the two indices. Combining the two indices into a single figure, the contour lines intersect at a single point. The horizontal and vertical coordinates of this intersection point correspond to the crack parameters S and P, providing precise information about the crack’s depth and location. Similarly, other crack parameters can be determined using a similar approach, as depicted in Figure 10a–d. By utilizing these contour lines and their intersections, researchers can accurately identify and retrieve various crack parameters, enhancing the effectiveness of crack detection and characterization in cantilever beams.

## 6. Wavelet Transform

A wavelet is a function that oscillates rapidly and has a short duration. Certain requirements, including compact support, admissibility, and vanishing moments, must be met by the Fourier transform of the system. These characteristics guarantee that the wavelet is confined in the time and frequency domains, which makes it advantageous for jobs involving signal processing and analysis. The wavelet transform allows for analyzing signals at various scales or resolutions, offering useful information about time and frequency.

The Continuous Wavelet Transform (CWT) is employed for analyzing spatial domain signals in beam structures to detect damages, especially closed cracks, and the mathematical expression of CWT is as follows:

(3) $${Wf}_{s,u}=\frac{1}{\sqrt{s}}\int_{-\infty }^{\infty }f(t)\psi^{*}\left(\frac{t-u}{s}\right)dt$$

where $\psi^{*}(t)$ is the complex conjugate of the ψ(t) and u and s > 0 are the translation and scale parameters, respectively. According to [39], the maximizing wavelet coefficients were used and we adapted the relative procedure for closed crack detection in a beam. The separation measure between the signal and wavelet is discussed, emphasizing their correlation. These insights provide a basis for utilizing CWT for damage detection in structures, with a primary focus on identifying subtle changes in dynamic behavior associated with minor damage, where traditional methods may lack effectiveness. The article underscores the significance of selecting an appropriate with minor damage, where traditional methods may lack effectiveness. The article underscores the significance of selecting an appropriate mother wavelet and achieving a balance in the number of vanishing moments for successful damage detection. The proposed technique is exemplified through its application to a cantilever beam model, showcasing its potential for damage detection.

### Wavelet Transform of RMS Signals

The root mean square (RMS) is a measure of the average power of a signal. It is obtained by taking the square root of the mean of squared values of the signal. The RMS value represents the magnitude of the signal’s fluctuation.

The wavelet transform is a widely used method in time-frequency analysis and signal processing. It enables us to break down a signal into several frequency components and examine the distribution of these components across time. Calculating the Root Mean Square (RMS) value is one technique. Analyzing the Root Mean Square (RMS) value of the wavelet coefficients is a typical technique for damage detection when applying wavelet transforms.

Using a collection of wavelet functions, the wavelet transform breaks down a signal into several scales. Comparing the RMS values of the wavelet coefficients before and after any damage has occurred is the principle behind damage detection. The presence of damage may be indicated if the RMS values at particular scales show considerable changes. We used Equation (4) to calculate the RMS value.

(4) $$RMS={\left(\sum_{i=1}^{N}\frac{{qi\left(t\right)}^{2}}{N}\right)}^{\frac{1}{2}}$$

where N denotes the total number of data points in the set, specifically in the displacement versus time graph, and *qi(t)* represents the displacement at the *i*th time point.

Table 4 lists different cases for the crack locations and depths of the beam.

**Effects of Crack Depth**

The damage depth can significantly affect the outcomes of wavelet transform analysis utilizing the RMS (Root Mean Square) approach. These fluctuations in RMS values can reveal important details regarding the type and severity of the signal’s damage. The RMS graph shows a straight-line pattern that represents a linear relationship between the RMS values and the associated node when a beam is undamaged.

**Figure 13:**
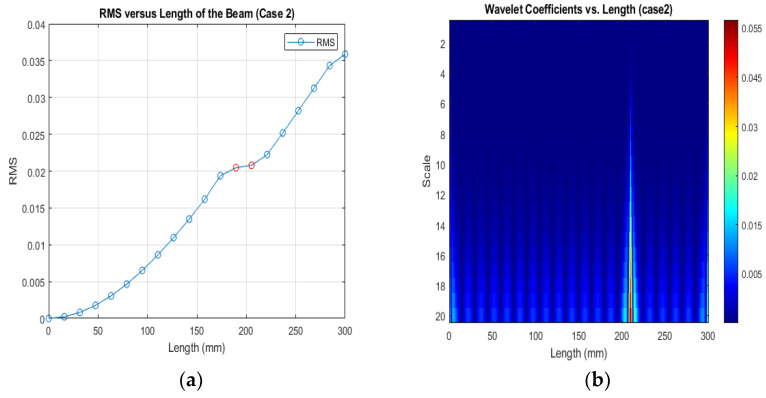
(**a**) RMS values under damage; (**b**) the wavelet coefficients for the beam under damage case 2.

**Figure 14:**
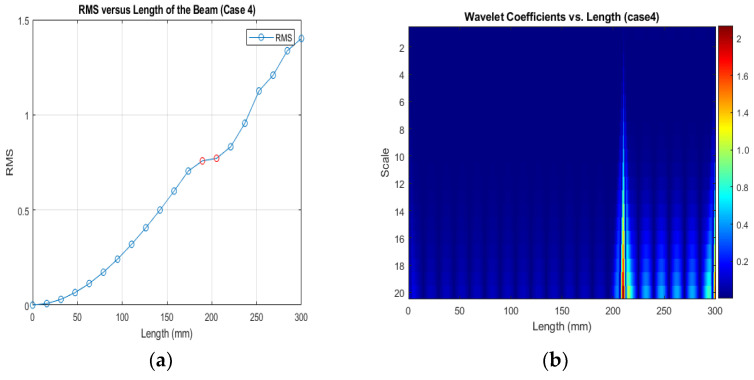
(**a**) RMS values under damage; (**b**) the wavelet coefficients for the beam under damage case 4.

**Figure 15:**
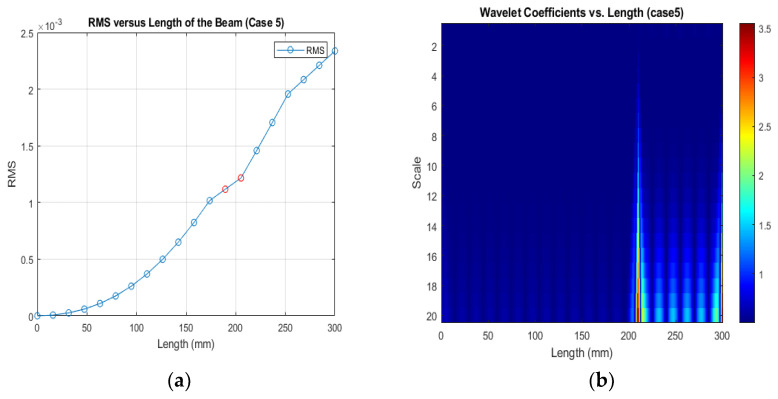
(**a**) RMS values under damage Case 5; (**b**) the wavelet coefficients for the beam under damage case 5.

Figure 13, Figure 14 and Figure 15 show that different damage depths affect the results of the RMS signal in the wavelet transform. The red dots indicate how the crack depth affect in the specified crack location. The presence of discontinuities in the RMS versus node graph is a strong indicator of crack occurrence in the beam. When a crack forms at a specific location along a beam, it introduces irregularities in the RMS values. These irregularities manifest as sudden changes in the RMS signal at the node where the crack is present. As the crack affects the structural integrity of the beam, it creates stress concentrations and alters the vibration characteristics, resulting in varying RMS values at different nodes. These distinct changes in the RMS signal offer valuable information for the detection of cracks in the beam.

**Effects of Crack Location**

The wavelet transform employing RMS can offer helpful insights into the location of damage within a structure. Unusual behaviors indicating damage can be identified by examining the variations in the RMS values at various nodes of the structure.

**Figure 16:**
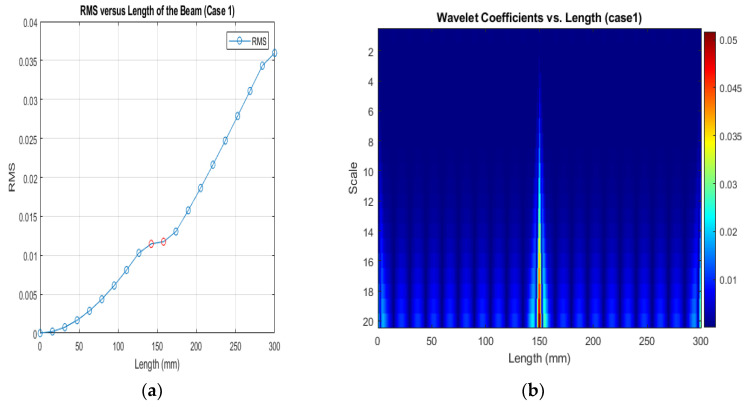
(**a**) RMS values under damage Case 1; (**b**) the wavelet coefficients for the beam under damage case 5.

**Figure 17:**
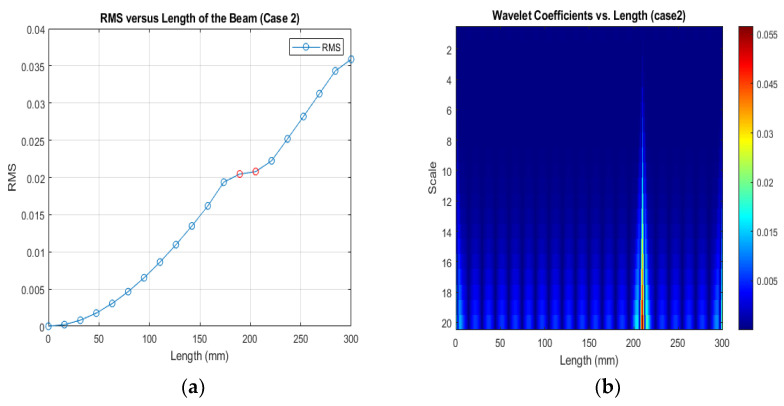
(**a**) RMS values under damage Case 2; (**b**) the wavelet coefficients for the beam under damage case 2.

**Figure 18:**
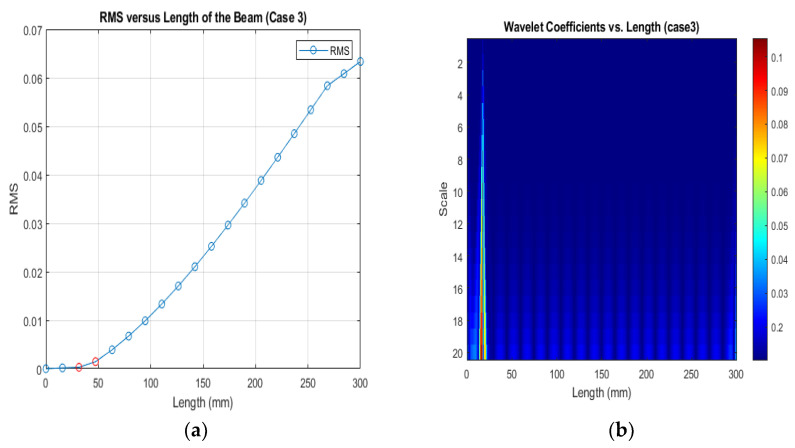
(**a**) RMS values under damage Case 3; (**b**) the wavelet coefficients for the beam under damage case 3.

Figure 16, Figure 17 and Figure 18 show that different damage locations affect the results of the RMS signal in the wavelet transform. Analyzing the RMS versus node graphs for different crack locations will highlight the unique response patterns at specific nodes. Different crack locations will lead to distinct node locations with irregularities that show in the red dot, aiding in detecting the crack’s position along the beam.

Based on the above results, when the crack location is far from the fixed end of a cantilever beam, the displacement decreases. This is because the structural stiffness near the fixed end is higher, and a crack farther away has a comparatively smaller impact on overall flexibility. As a result, the displacement decreases due to the reduced influence of the crack on the beam’s deformation behavior. In the case of the crack depth effect, when the crack depth increases in a cantilever beam with the crack located far from the fixed end, the displacement increases. An increase in crack depth leads to a more substantial reduction in structural stiffness, making the beam less resistant to deformation. As a result, the displacement tends to increase with greater crack depth, indicating a heightened impact on the overall flexibility and behavior of the beam. In conclusion, the outcomes of the wavelet transform utilizing RMS depend critically on the location of the damage. The RMS values at various nodes can offer important details regarding the existence and severity of damage in a beam. However, it is crucial to consider the precise location of the damage and study the distribution of the RMS values throughout the nodes to accomplish accurate and reliable damage detection.

## 7. Comparison of the Phase Diagram Results with Wavelet Transform RMS Signal

According to the results discussed above, a comparison between the phase diagram and the wavelet coefficients using an RMS signal reveals that both methods provide valuable insights into the behavior of the beam. The findings indicate that the crack location and damage depth each have their effects on the beam’s behavior. Both methods offer a comprehensive understanding of the behavior of the cracked beam. This observation suggests that both approaches provide unique advantages in understanding and characterizing the beam’s response to cracks. The fact that both methods lead to the same conclusion further strengthens the validity of the results. It implies that the combined analysis of velocity-displacement diagrams and wavelet coefficients yields a comprehensive understanding of the beam’s condition.

The comparative study of wavelet coefficients and phase diagrams for closed cracking detection in beams sheds light on how structural health monitoring techniques are developing. The research presented emphasizes the importance of advanced techniques in identifying and characterizing cracks, acknowledging the limitations of traditional methods, and addressing the challenges posed by complex structural environments. The investigation of vibration properties in cracked beams using phase diagrams and wavelet coefficients shows promise for a more complex and dynamic crack detection method. The suggested approach provides a potential means of identifying the position and depth of cracks by taking advantage of the geometric properties of orbital inclusion areas in phase diagrams. This method increases the possibilities for crack identification, especially when dealing with barely visible cracks.

## 8. Conclusions

This study employed various approaches to examine the effects of crack parameters on the phase diagram of a cantilever beam with a closed crack. A method for determining crack parameters based on the geometric characteristics of the phase diagram is described. The study involved creating models of both an intact cantilever beam and a damaged beam with a closed crack. It was discovered that the harmonic vibration response is sensitive and may identify the presence of a barely visible crack by comparing the acceleration time-history and phase spectrum acquired through harmonic excitation at various frequencies. The results demonstrated that the phase diagram displays a single-loop shape for a cantilever beam excited at the principal resonance frequency. The total phase diagram area grows slightly as the crack moves gradually away from the free end or as the crack depth rises. Phase diagrams for single-loop and double-loop excitations of the beam’s super-harmonic resonance frequency be seen.

The area of the phase diagram is determined using the pixel method. Two sets of indexes are defined for different types of responses. In the case of a single-period response, indexes are defined. Contour maps are created to visualize the variations of these index groups concerning the crack position and depth. By examining the contour maps, the position and depth of the crack can be obtained from the intersection point of the contour lines. This intersection point is the key indicator for accurately identifying the crack parameters. The crack position and depth values can be determined based on their coordinates at the intersection point.

The sensitivity of the suggested damage detection index and the usefulness of the contact model in closed crack modeling are verified through numerical simulation. With the increase in crack location and depth, the indexes gradually increase. Moreover, when the crack parameters are the same, the excitation force has little effect on the defined parameters. For future research, we suggest analyzing the effects of multiple cracks and finding the capability of reducing the vibration amplitude of the cracked beam. The analysis of wavelet coefficients and the velocity-displacement diagram confirms that both crack location and depth significantly affect the behavior of the cantilever beam. The agreement between the two methods strengthens the reliability of the results, providing a comprehensive assessment of the beam’s condition and structural integrity. For future works, it is crucial to build upon this foundation by expanding numerical investigations to a wider range of structural configurations and loading conditions. The incorporation of diverse materials and scenarios will enhance the robustness and reliability of crack detection methods. Additionally, efforts should be directed toward standardizing numerical simulation protocols and methodologies, ensuring consistency and comparability across different studies.

## Figures and Tables

**Table 1:** Material properties of Mild steel [35].

Material Properties	Value	Unit
Elastic modulus (E)	206.8	GPa
Poisson Ratio	0.3	-
Density	7850	kg/m^3^

**Table 2:** Values of A(LR) and A(UD) under different crack depths.

S	P	A(UD)	A(LR)
0.3	0.5	0.8031	0.9648
0.5	0.5	0.8320	1.0001
0.5	0.7	0.9983	1.0051

**Table 3:** Values of A(LR) and A(UD) under different crack locations.

S	P	A(UD)	A(LR)
0.1	0.5	0.1022	0.6865
0.3	0.1	0.2322	0.6899
0.3	0.7	0.8831	0.9583

**Table 4:** Different cases for the crack locations and depths of the beam.

Scenario	Crack Position (Lc/L)	Damage Severity (a/h)
Case 1	0.5	0.1
Case 2	0.7	0.1
Case 3	0.06	0.1
Case 4	0.7	0.3
Case 5	0.7	0.5

## Data Availability

Data are contained within the article.
